# Supplementary material for: Shadow imaging for panoptical visualization of brain tissue in vivo
Source: Nat Commun. 2023 Oct 12;14:6411. doi: 10.1038/s41467-023-42055-2 (PMC10570379; doi:10.1038/s41467-023-42055-2)
Supplement: Supplementary file 1 — Supplementary Information [file 41467_2023_42055_MOESM1_ESM.pdf]

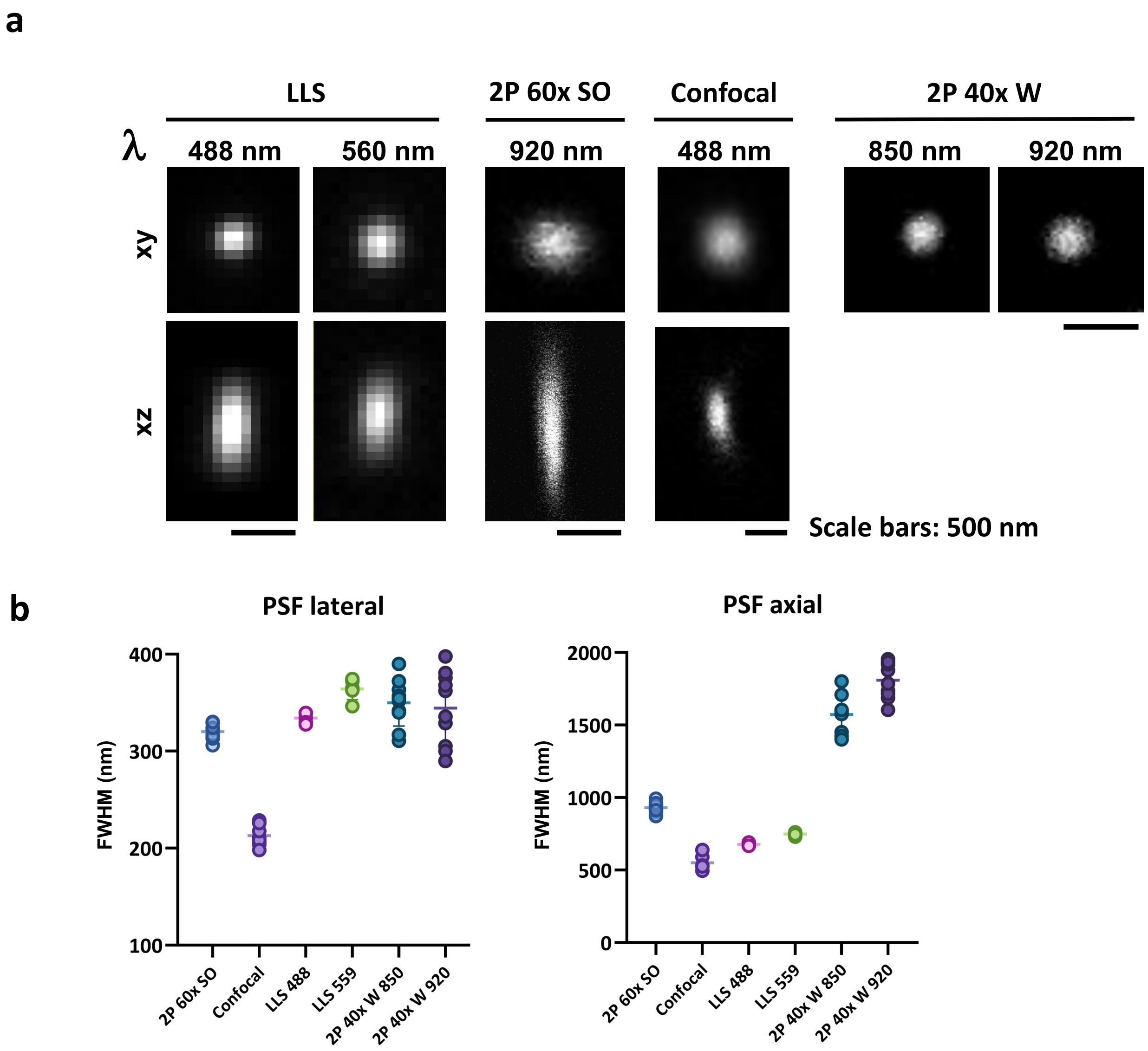

Supplementary Figure 1: PSF measurements of various microscopy modalities.

a Representative PSFs in both lateral and axial dimensions captured with fluorescent beads in all four microscopes. **b** Quantification of PSF measurements laterally and axially in all optical modalities. W: water objective; SO: silicon-oil objective.

Timecourse of fluorescence signals averaged over FOV

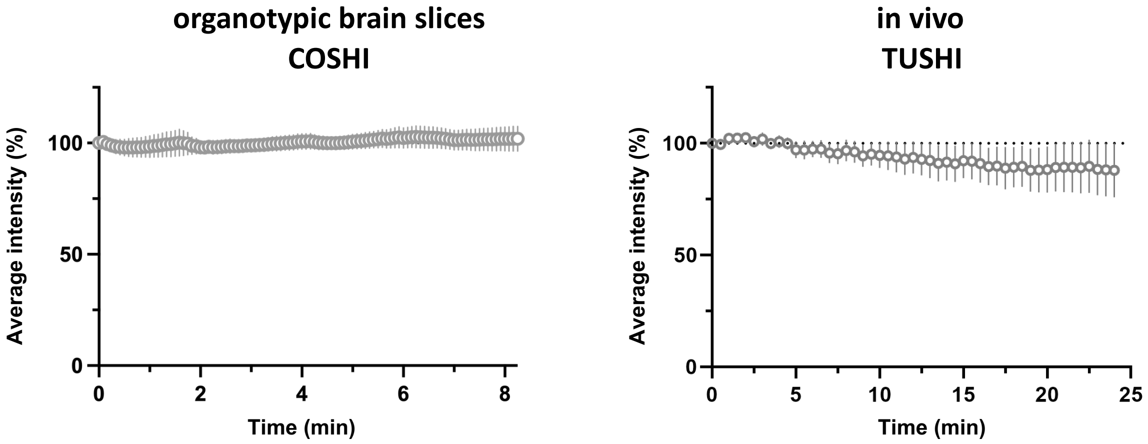

Supplementary Figure 2: Bleaching/clearance of fluorescent dye.

Timecourse of fluorescence signal averaged over entire FOV during repeated imaging in organotypic slices for COSHI (N = 6) and *in vivo* for TUSHI (N = 8).

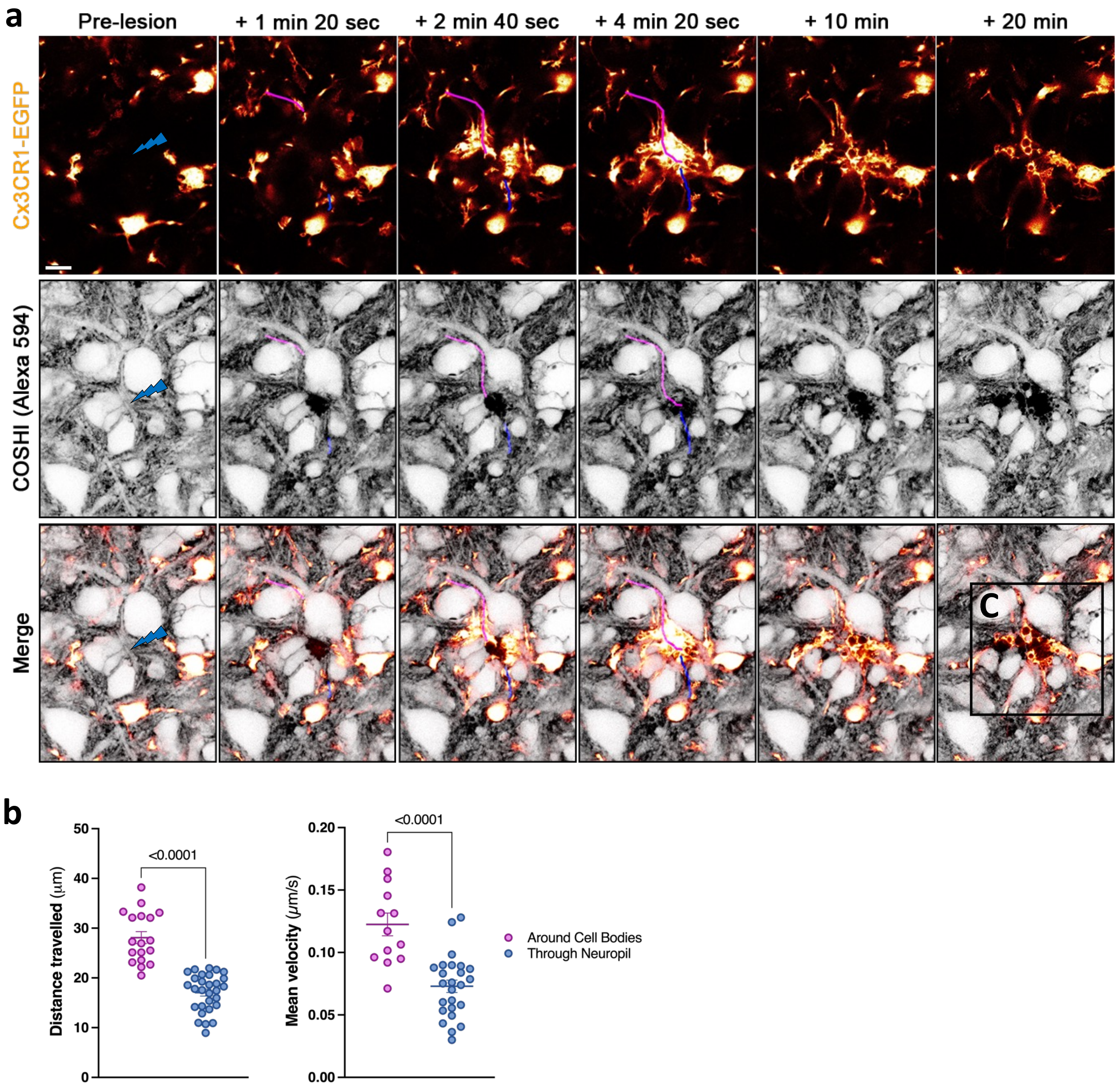

Supplementary Figure 3: Microglial dynamics in the context of their cellular environment.

a Representative time series of labeled microglia cells (CX3CR1-EGFP) and confocal shadow images (Alexa Fluor 594, 200 mM) along with merged channels showing their processes travelling towards a laser lesion (blue bolt; expanded from **Fig. 2**; see also **SI Movie 6**). Scale bar, 10 mm. Inset in bottom right highlights the presence of microglial phagocytosis at the lesion site (see **SI Fig. 2C**). **b** Microglial processes travelled further with greater mean velocity (average of 5 consecutive frames between t = 60 s and t = 140 s) when approaching a lesion and circumventing an impeding cell body, relative to processes that travelled directly through neuropil (n = 14 around cell bodies, n = 29 through neuropil, from 8 lesions). Unpaired t-tests.

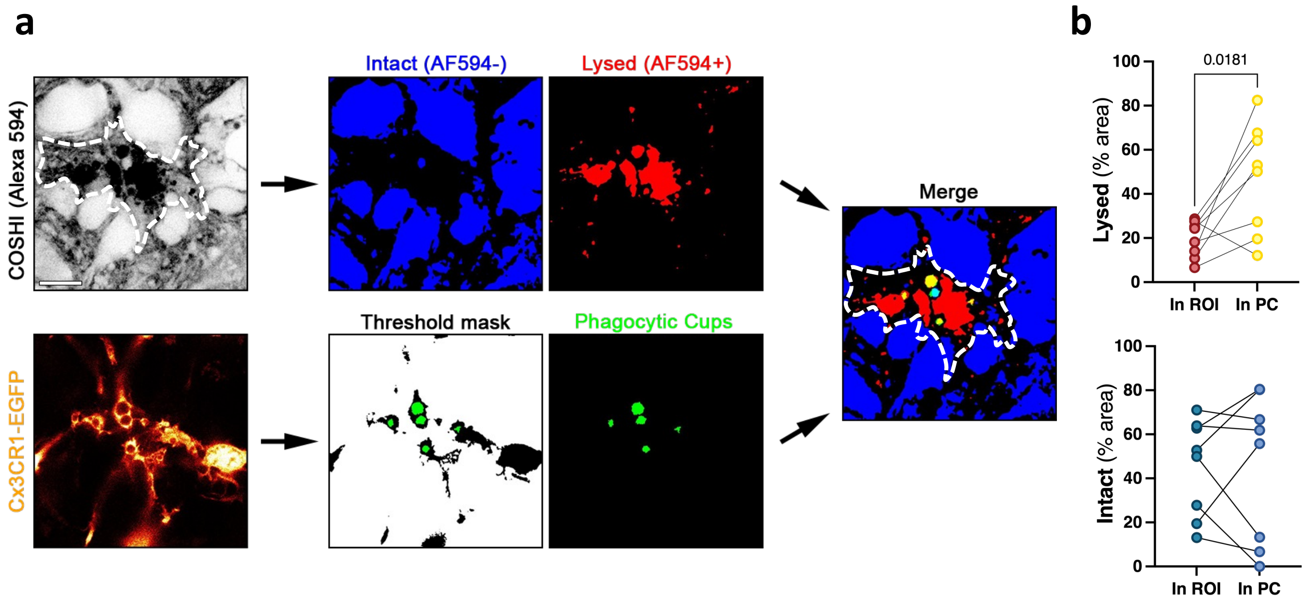

Supplementary Figure 4: Phagocytosis by microglia after laser lesion.

a Flowchart for microglial phagocytic triage analysis. Intact (dye-negative; blue) and lysed (dye-positive; red) cellular structures were identified by thresholding inverted COSHI time-stacks based on intensities of >70 % and <2 % of pixels, respectively. The developing lesion area (white dashed line) was an ROI containing lysed or blebbing/dysmorphic intact structures. Putative phagocytic cups were identified in CX3CR1-EGFP time-stacks by subtracting one binarized copy of the time-stack from another (after Fill Holes FIJI function), resulting in signal only where "holes" were present in the CX3CR1-EGFP images (green). Next, the 3D Object Analysis FIJI function was used to favor circular structures in the size range of phagocytic cups. The ROIs (green) were then redirected to the intact (blue) or lysed (red) COSHI time-stacks. A phagocytic cup was considered positive for intact or lysed structures if the median value of the ROI contents was non-zero in either the dye-negative, dye-positive, or both channels, respectively. In the merged image, phagocytic cups containing intact or lysed structures are seen as cyan or yellow, respectively. See Methods for details. Scale bars, 10 µm. **b** Significant enrichment of lysed tissue in phagocytic cups was demonstrated by comparing the percentage of developing lesion areas (in ROI) relative to the percentage of phagocytic cups (in PC) containing lysed structures across each time series (n = 8 lesions, paired t-tests). Enrichment of intact structures was not observed. See Methods for additional details.
