# Supplementary material for: Shadow imaging for panoptical visualization of brain tissue in vivo
Source: Nat Commun. 2023 Oct 12;14:6411. doi: 10.1038/s41467-023-42055-2 (PMC10570379; doi:10.1038/s41467-023-42055-2)
Supplement: Supplementary file 18 — Reporting Summary [file 41467_2023_42055_MOESM18_ESM.pdf]

nature portfolio

Corresponding author(s): U. Valentin Nägerl

Last updated by author(s): 04/09/2023

Reporting Summary

Nature Portfolio wishes to improve the reproducibility of the work that we publish. This form provides structure for consistency and transparency in reporting. For further information on Nature Portfolio policies, see our Editorial Policies and the Editorial Policy Checklist.

Statistics

For all statistical analyses, confirm that the following items are present in the figure legend, table legend, main text, or Methods section.

n/a	Confirmed	
☐	☒	The exact sample size (*n*) for each experimental group/condition, given as a discrete number and unit of measurement
☐	☒	A statement on whether measurements were taken from distinct samples or whether the same sample was measured repeatedly
☐	☒	The statistical test(s) used AND whether they are one- or two-sided<br>*Only common tests should be described solely by name; describe more complex techniques in the Methods section.*
☒	☐	A description of all covariates tested
☐	☒	A description of any assumptions or corrections, such as tests of normality and adjustment for multiple comparisons
☐	☒	A full description of the statistical parameters including central tendency (e.g. means) or other basic estimates (e.g. regression coefficient) AND variation (e.g. standard deviation) or associated estimates of uncertainty (e.g. confidence intervals)
☐	☒	For null hypothesis testing, the test statistic (e.g. *F*, *t*, *r*) with confidence intervals, effect sizes, degrees of freedom and *P* value noted<br>*Give P values as exact values whenever suitable.*
☒	☐	For Bayesian analysis, information on the choice of priors and Markov chain Monte Carlo settings
☒	☐	For hierarchical and complex designs, identification of the appropriate level for tests and full reporting of outcomes
☐	☒	Estimates of effect sizes (e.g. Cohen's *d*, Pearson's *r*), indicating how they were calculated

Our web collection on statistics for biologists contains articles on many of the points above.

Software and code

Policy information about availability of computer code

Data collection	Image acquisition was performed by the commercial software of the respective microscopes used in the study, as detailed in the manuscript.
Data analysis	Images were analyzed using ImageJ and data were plotted using GraphPad Prism.

For manuscripts utilizing custom algorithms or software that are central to the research but not yet described in published literature, software must be made available to editors and reviewers. We strongly encourage code deposition in a community repository (e.g. GitHub). See the Nature Portfolio guidelines for submitting code & software for further information.

Data

Policy information about availability of data

All manuscripts must include a data availability statement. This statement should provide the following information, where applicable:

- Accession codes, unique identifiers, or web links for publicly available datasets
- A description of any restrictions on data availability
- For clinical datasets or third party data, please ensure that the statement adheres to our policy

The datasets generated and/or analyzed during the current study are available from the corresponding author on request.

Human research participants

Policy information about studies involving human research participants and Sex and Gender in Research.

Reporting on sex and gender	*Use the terms sex (biological attribute) and gender (shaped by social and cultural circumstances) carefully in order to avoid confusing both terms. Indicate if findings apply to only one sex or gender; describe whether sex and gender were considered in study design whether sex and/or gender was determined based on self-reporting or assigned and methods used. Provide in the source data disaggregated sex and gender data where this information has been collected, and consent has been obtained for sharing of individual-level data; provide overall numbers in this Reporting Summary. Please state if this information has not been collected. Report sex- and gender-based analyses where performed, justify reasons for lack of sex- and gender-based analysis.*
Population characteristics	*Describe the covariate-relevant population characteristics of the human research participants (e.g. age, genotypic information, past and current diagnosis and treatment categories). If you filled out the behavioural & social sciences study design questions and have nothing to add here, write "See above."*
Recruitment	*Describe how participants were recruited. Outline any potential self-selection bias or other biases that may be present and how these are likely to impact results.*
Ethics oversight	*Identify the organization(s) that approved the study protocol.*

Note that full information on the approval of the study protocol must also be provided in the manuscript.

Field-specific reporting

Please select the one below that is the best fit for your research. If you are not sure, read the appropriate sections before making your selection.

[x] Life sciences [ ] Behavioural & social sciences [ ] Ecological, evolutionary & environmental sciences

For a reference copy of the document with all sections, see nature.com/documents/nr-reporting-summary-flat.pdf

Life sciences study design

All studies must disclose on these points even when the disclosure is negative.

Sample size	The study reports on the development of a new microscopy method, presenting technical data on the proof-of-concept of the approach (e.g., image metrics like signal-to-noise ratios and spatial resolution) and biological data to validate its utility for several neurobiological applications discussed in the manuscript. Concerning the technical data, the experimental conditions were optimized until sufficient image quality and repeatability were assured. Concerning the biological data, the experiments were repeated in multiple samples (prepared from different animals) to capture the biological variability, and be able to detect statistically significant differences and/or correlations between the experimental read-outs. For the analysis of microglia, sample size was chosen based on similar analyses in the literature (Davalos et al., Nature Neuroscience, 2005; Avignone et al., J Neuroinflammation, 2015; Stoyanov et al., Glia, 2020). For the analysis of PVS and blood vessel sizes, we used sample size as in similar studies (J. Steinman et al., PlosOne 2017; N. Raicevic et al., Fluids Barriers CNS, 2023). For in vivo VF measurements, we used a 3 times larger sample size compared to VF estimation in vitro (J. Tønnesen et al., Cell, 2018).
Data exclusions	Data exclusion is not a relevant category for method developments. The optical hardware, labeling, sample preparation, image acquisition and analysis were optimized until the performance of the new method was deemed satisfactory and reliable enough to conclude successful proof-of-concept. For the biological experiments, no data were excluded.
Replication	The technical and biological experiments were repeated multiple times yielding similar results, as spelled out in the paper. The number of replication aligns with similar studies in our field, ensuring scientific reliability, while keeping the number of animals used in our study low for ethical reasons.
Randomization	Not applicable.
Blinding	Not applicable.

Reporting for specific materials, systems and methods

We require information from authors about some types of materials, experimental systems and methods used in many studies. Here, indicate whether each material, system or method listed is relevant to your study. If you are not sure if a list item applies to your research, read the appropriate section before selecting a response.

Materials & experimental systems

n/a	Involved in the study
x	Antibodies
x	Eukaryotic cell lines
x	Palaeontology and archaeology
	x Animals and other organisms
x	Clinical data
x	Dual use research of concern

Methods

n/a	Involved in the study
x	ChIP-seq
x	Flow cytometry
x	MRI-based neuroimaging

Animals and other research organisms

Policy information about studies involving animals; ARRIVE guidelines recommended for reporting animal research, and Sex and Gender in Research

Laboratory animals	C57Bl/6J wild-type (3-5 days old and 3-5 months old), CX3CR1-EGFP (3-5 days old) and immunodeficient Rag 2 Gamma C-/- mice (3-4 months-old) of both sexes were used in this study.
Wild animals	Not applicable.
Reporting on sex	Animals of both sexes were used, and sex was not considered in the study.
Field-collected samples	Not applicable.
Ethics oversight	Animal handling and experimental procedures were in accordance with the European Union and CNRS institutional guidelines for the care and use of laboratory animals (Council directive 2010/63/EU) and approved by the Institutional Animal Care and Use Committee at the University of Bordeaux, France (DAP 2019031909389750_v7).

Note that full information on the approval of the study protocol must also be provided in the manuscript.
