# Supplementary material for: Shadow imaging for panoptical visualization of brain tissue in vivo
Source: Nat Commun. 2023 Oct 12;14:6411. doi: 10.1038/s41467-023-42055-2 (PMC10570379; doi:10.1038/s41467-023-42055-2)
Supplement: Supplementary file 19 — Description of Additional Supplementary Files [file 41467_2023_42055_MOESM19_ESM.pdf]

Description of Additional Supplementary Files:

Supplementary Movie 1: COSHI z stack in organotypic brain slice

Supplementary Movie 2: COSHI z stack in organotypic brain slice

Supplementary Movie 3: COSHI z stack in organotypic brain slice

Supplementary Movie 4: COSHI time series

Supplementary Movie 5: COSHI z stack showing EGFP-labeled microglia cell

Supplementary Movie 6: Segmented neuropil around EGFP-labeled microglia cell

Supplementary Movie 7: COSHI time series showing microglial response to laser lesion

Supplementary Movie 8: LISHI z stack with Calcein

Supplementary Movie 9: LISHI time series of z stack

Supplementary Movie 10: LISHI z stack with Alexa Fluor 568

Supplementary Movie 11: LLS of iGluSnFR signals

Supplementary Movie 12: LISHI around iGluSnFR signals

Supplementary Movie 13: LLS of GCaMP6f signals

Supplementary Movie 14: LISHI around GCaMP6f signals

Supplementary Movie 15: TUSHI z-stack in vivo

Supplementary Movie 16: TUSHI z stack showing YFP-labeled tumor cells in motor cortex in vivo
